# An HDL Proteomic Score Independently Predicts Incident Atherosclerotic Cardiovascular Disease

**DOI:** 10.1016/j.jacadv.2025.101844

**Published:** 2025-06-25

**Authors:** Olle Melander, Anand Rohatgi, Judy Z. Louie, Timothy S. Collier, Michael J. McPhaul, Fahim Abbasi

**Affiliations:** a Department of Clinical Sciences Malmö, Lund University, Malmö, Sweden; b University of Texas Southwestern Medical Center at Dallas, Dallas, Texas, USA; c Quest Diagnostics Nichols Institute, San Juan Capistrano, California, USA; d Quest Diagnostics Cardiometabolic Center of Excellence, Cleveland HeartLab, Cleveland, Ohio, USA; e Stanford University School of Medicine, Palo Alto, California, USA

**Keywords:** ApoA1, ASCVD, proteomics

High-density lipoprotein (HDL) proteomic composition using a panel of 5 apolipoproteins within HDL (apoA1, apoC1, apoC2, apoC3, and apoC4), referred to as pCAD, is associated with cholesterol efflux capacity, a key HDL function.^1^^,^^2^ pCAD has been associated with angiographic evidence of obstructive coronary artery disease^3^ but whether pCAD can improve prediction of incident atherosclerotic cardiovascular disease (ASCVD) in a primary prevention population remains unknown.

**What is the clinical question being addressed?**

Does pCAD predict incident ASCVD in participants free from ASCVD at baseline?

**What is the main finding?**

pCAD score was associated with incident ASCVD: in the full model adjusted for follow-up time and TRFs, including HDL-C, pCAD remained significantly associated with incident ASCVD.

We conducted a nested case-control−matched study that derived from the Malmö Preventive Project. This population-based prospective cohort study^4^ enrolled 18,240 participants with baseline examination between 2002 and 2006 and follow-up time to December 2019 (66% males, median [IQR] age 70 [67-73] years and 13.3 years of follow-up time). We randomly selected 200 participants who developed incident ASCVD events during follow-up and 200 age- and sex-matched participants who did not. At baseline, none of the 400 participants had prevalent ASCVD. Incident ASCVD events were defined as first incident ASCVD, during a median (IQR) follow-up of 10 (5-14) years among the 400 analyzed subjects, that included fatal myocardial infarction or coronary heart disease, nonfatal myocardial infarction, coronary revascularization, and fatal or nonfatal stroke.

HDL apolipoproteins were measured by mass spectrometry in serum specimens collected from fasting patients during the baseline examination and the pCAD score was calculated using coefficients prespecified from prior studies.^2^^,^^3^ Fasting insulin and C-peptide were also measured by mass spectrometry and were used to calculate insulin resistance risk score (IRRS).^5^ The association between pCAD score and incident ASCVD status was assessed using conditional logistic regression adjusting for follow-up time in a crude model and for follow-up time and traditional risk factors (TRFs) including HDL cholesterol (HDL-C), total cholesterol, hypertension, smoking, and diabetes in a full model. The association between pCAD score and incident ASCVD status was also assessed using logistic regression models according to insulin resistance status defined as IRRS above or below median, indicating higher and lower insulin resistance, respectively. The differences in baseline variables between incident ASCVD cases and controls were assessed using the Wilcoxon signed rank test for continuous variables and the McNemar test for categorical variables.

Participants with incident ASCVD events had lower HDL-C levels, higher total cholesterol levels, lower proportion of smoking, shorter follow-up years, and higher pCAD score than those without incident ASCVD events (*P* < 0.05). Higher pCAD score was associated with incident ASCVD. In the full model adjusted for follow-up time and TRFs, pCAD was significantly associated with incident ASCVD (OR [95% CI] per 1 SD increment in pCAD, 1.90 [1.25-2.91], *P* = 0.003); whereas HDL-C was not (*P* = 0.50) (Figure 1A). Continuous Net Reclassification Index (NRI) (95% CI) for ASCVD improved by 24% (4.6% to 43%) (*P* = 0.015) when adding pCAD to TRFs. The NRI (95% CI) for up-classification among those with ASCVD events was 22% (8.5% to 36%), *P* = 0.001, whereas the corresponding NRI for down-classification among those without events was 2% (−12% to 16%), *P* = 0.78, suggesting that pCAD contributes mainly to up-classification of risk. When pCAD was added on top of a model with TRFs, the area under the receiver operating characteristic curve (95% CI) changed nonsignificantly from 0.85 (0.82-0.89) to 0.86 (0.83-0.90) (*P* = 0.16). The Integrated Discrimination Index was significant when pCAD was added on top of TRFs 0.019 (0.005-0.033) (*P* = 0.008). Finally, in a likelihood ratio test, the model with TRFs improved significantly when pCAD was added to the model (*P* = 0.001).

**Figure 1:**
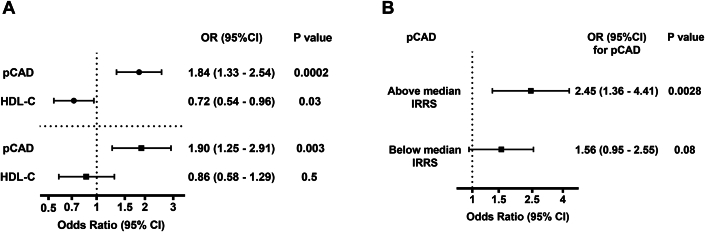
pCAD and Incident ASCVD (A) Association between pCAD and incident ASCVD depicted as OR (95% CI) for incident ASCVD per 1 SD increment in pCAD and HDL-C, adjusted for follow-up time (filled circles), adjusted for HDL-C (pCAD) (filled squares), total cholesterol, hypertension, smoking, diabetes, and follow-up time. (B) Association between pCAD and incident ASCVD according to insulin resistance status depicted as OR (95% CI) for incident ASCVD per 1 SD increment in pCAD in participants with IRRS above the median (higher insulin resistance) and below the median (lower insulin resistance), adjusted for age, sex, HDL-C, total cholesterol, hypertension, smoking, diabetes, and follow-up time. IRRS: median IRRS (11%). ASCVD = atherosclerotic cardiovascular disease; HDL-C = high-density lipoprotein cholesterol; IRRS = insulin resistance risk score.

The correlation between continues values of pCAD and IRRS was R = 0.36 (*P* < 0.0001). The interaction term between pCAD and IRRS strata (above vs below the median) was not significant (*P* = 0.12). pCAD (OR [95% CI] per 1 SD increment) was associated with incident ASCVD among participants with IRRS above the median (2.45 [1.36-4.41]) but not in those with IRRS below the median (1.56 [0.95-2.55]), after adjusting for TRFs, including HDL-C (Figure 1B).

Our results show that HDL-associated protein composition expressed as pCAD is associated with incident ASCVD independently of TRFs including HDL-C, suggesting a role of pCAD in risk stratification in the primary prevention setting. Larger studies are needed to replicate and validate this finding and to test if there is effect modification by insulin resistance.

## Funding support and author disclosures

This work was supported by the Swedish Heart and Lung Foundation (grant 20240352). Drs Louie, Collier, and McPhaul are current or former employees of, and may own stock in, Quest Diagnostics. Dr Rohatgi has received a research grant from CSL Behring and act as a consultant for HDL Diagnostics. All other authors have reported that they have no relationships relevant to the contents of this paper to disclose.
